# Understanding the dimorphic lifestyles of human gastric pathogen *Helicobacter pylori* using the SWATH-based proteomics approach

**DOI:** 10.1038/srep26784

**Published:** 2016-05-25

**Authors:** Mun Fai Loke, Chow Goon Ng, Yeespana Vilashni, Justin Lim, Bow Ho

**Affiliations:** 1 Department of Medical Microbiology, Faculty of Medicine, University of Malaya, Kuala Lumpur, Malaysia; 2 Department of Microbiology and Immunology, Yong Loo Lin School of Medicine, National University of Singapore, Singapore; 3 AB SCIEX Ltd, 10 Biopolis Rd, Singapore 138670, Singapore

## Abstract

*Helicobacter pylori* may reside in the human stomach as two morphological forms: the culturable spiral form and the viable but non-culturable (VBNC) coccoid form. This bacterium transforms from spiral to coccoid under *in vitro* suboptimal conditions. However, both spiral and coccoid have demonstrated its infectivity in laboratory animals, suggesting that coccoid may potentially be involved in the transmission of *H. pylori*. To determine the relevance of the coccoid form in viability and infectivity, we compared the protein profiles of *H. pylori* coccoids obtained from prolonged (3-month-old) culture with that of 3-day-old spirals of two *H. pylori* standard strains using SWATH (Sequential Window Acquisition of all Theoretical mass spectra)-based approach. The protein profiles reveal that the coccoids retained basal level of metabolic proteins and also high level of proteins that participate in DNA replication, cell division and biosynthesis demonstrating that coccoids are viable. Most interestingly, these data also indicate that the *H. pylori* coccoids possess higher level of proteins that are involved in virulence and carcinogenesis than their spiral counterparts. Taken together, these findings have important implications in the understanding on the pathogenesis of *H. pylori*-induced gastroduodenal diseases, as well as the probable transmission mode of this bacterium.

*Helicobacter pylori* is a Gram-negative microaerophilic bacterium strongly associated with gastroduodenal diseases ranging from chronic gastritis, peptic ulcers to gastric adenocarcinoma and MALT lymphomas^1^. The prevalence of *H. pylori* is about 50% worldwide and up to 90% in developing countries^2^, yet the mode of its transmission remains not well established to date. This bacterium can reside in the human stomach as two morphological forms: the spiral and the viable but non-culturable coccoid (VBNC) forms^3^,^4^. To date, there is only one report of *H. pylori* coccoid form reverting to spiral form under *in vitro* conditions^5^. However, we failed to resuscitate the coccoid form using method reported. Nevertheless, non-culturable coccoid of *H. pylori* has been shown to revert back to spiral form in mice stomach^6^.

Earlier study by Hua and Ho^7^ had shown that ageing coccoid cultures produce alkaline phosphatase, acid phosphatase, leucine arylamidase (leucyl aminopeptidase; PepA) and naphthol-AS-β 1-phosphohydrolase similar to the exponential cultures and remain genetically unaltered, suggesting their viability^7^. Indeed, both forms are able to colonize mice, cause gastritis and stimulate immune response^6^,^8^. The two forms can adhere to the human gastric epithelial cells (such as GES-1) with the spiral form induces higher pro-apoptotic proteins (e.g. Fos, Gadd45a and Myc) expression while the latter induces higher anti-apoptotic protein (survivin)^9^. Survivin is frequently linked to the development of cancer suggesting that coccoid form may have a role to play in gastric carcinogenesis.

The spiral form has been shown to co-exist with unaltered or variously damaged mucous cells whereas coccoid is closely associated with severely damaged gastric mucous cells^3^. While spiral *H. pylori* has been reported to be associated with chronic active gastritis, coccoid *H. pylori* is more frequently associated with chronic inactive gastritis in symptomatic adult patients^4^. Symptomatic pediatric patients and pediatric patients with recurrent epigastric pain have 4-fold higher seroprevalence for coccoid antigen compared to that for spiral antigen indicating a possible infective role of the coccoid form of *H. pylori* in these patients^10^.

*H. pylori* undergoes morphological conversion from spiral to coccoid when cultured under mild sub-optimal growth conditions^11^. These conditions include aerobiosis^12^,^13^, acidic and alkaline pH^12^,^13^,^14^, high temperature^15^, extended incubation^10^,^13^, treatment with a proton pump inhibitor^13^ and treatment with antibiotics^16^. Coccoid form of *H. pylori* has been shown to preserve its RNA, DNA and structural components for at least 3 months^16^. Extended incubation is gradual and least stressful for the bacterium as compared to other methods of inducing formation of coccoids. Furthermore, this approach is probably more relevant for *H. pylori* as the organism is known to colonize the human stomach for life unless eradicated^17^. *H. pylori* coccoid form expresses high-molecular weight antigens that are not expressed by the spiral form^18^ indicating that *H. pylori* coccoids that are formed under prolonged culture may still be viable and immunogenic. However, no coccoid-specific protein has been identified using two-dimensional gel electrophoresis^19^ limiting our current understanding of the coccoid lifestyle of *H. pylori*. The lack of known coccoid-specific proteins also hinders the development of *H. pylori* coccoid-specific detection assays. The main hurdles of using gel-based to analyze the proteome of *H. pylori* coccoid sample include low protein content and the accumulation of metabolic or degradation byproducts that can interfere with gel-electrophoresis. Thus, there is a need to apply a more sensitive and gel-free method for proteome comparison between *H. pylori* spiral and coccoid forms. In this study, liquid chromatography-mass spectrometry (LC-MS) with the capability to efficiently differentiate the two proteomes was used. To address this issue, we compared the protein compositions of 3-day old spiral cells in liquid cultures to that of 3-month old coccoid cells grown in stationary liquid culture of two strains of *H. pylori,* NCTC 11637 and J99, using the LC-MS-based SWATH (Sequential Window Acquisition of all Theoretical mass spectra) approach. *H. pylori* NCTC 11637 is the type strain^20^ while J99 is one of the earliest strains that were fully sequenced^21^. Both *H. pylori* strains have been used in laboratory experiments. The SWATH strategy makes use of the fragment ion spectral libraries to mine the complete fragment ion maps generated based on a data-independent acquisition method. The method enables the detection of large number of proteins reproducibly at near quantitative accuracy in a single measurement^22^. Unlike traditional multiple reaction monitoring (MRM)-based quantitation method, where the target protein has to be known before data acquisition during to method setup. SWATH-MS is for unbiased untargeted quantitation of proteins using MS. This provides an added advantage for this study as we are interested to understand the global differential expression using a non-label strategy. Furthermore, this data-acquisition method will be most productive for future retrospective mining of quantitative data when potential target candidates of interest have been identified.

## Results and Discussion

**Figure 1:**
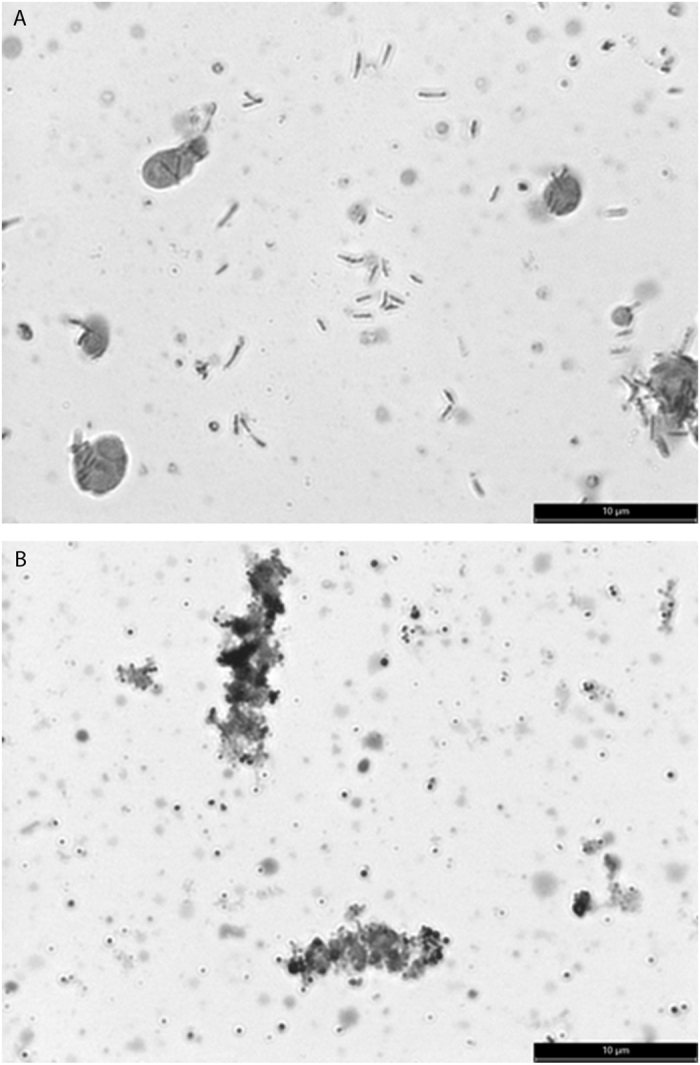
Direct wet-mount micrograph of (**A**) 3-day old spiral and (**B**) 3-month old coccoid appeared in clumps (Magnification: 1000X).

In this study, *H. pylori* coccoids from 3-month-old culture of two *H. pylori* strains were harvested for proteome analysis in comparison with the spirals from 3-day-old cultures (Fig. 1). An average of 914 proteins per sample was identified from the spiral and coccoid protein extracts of *H. pylori* NCTC 11637 and J99 strains with <1% global protein false discovery rate and 99% confidence threshold (Supplementary data). Proteins showing statistical significant difference by Student’s *t*-test (p-value < 0.05) using MarkerView software were annotated and classified based on KEGG (Kyoto Encyclopedia of Genes and Genomes) Orthology (KO) using STRING version 9.1 (www.string-db.com)^23^. For both *H. pylori* strains, 81 proteins were significantly different (p-value < 0.05) with coccoid/spiral fold-change <1.0 (Supplementary Table S1). On the other hand, 66 proteins showed significant difference in expression (p-value < 0.05) for coccoid forms in comparison to their spiral counterparts with coccoid/spiral fold-change >1.0 (Supplementary Table S2).

### Metabolism

**Figure 2:**
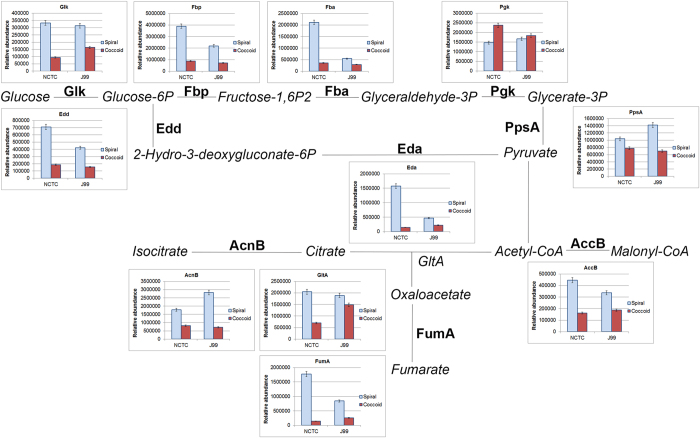
Core carbon metabolism pathway. Glucokinase (Glk), fructose-1,6-biphosphate (Fbp), fructose-biphosphate aldolase (Fba), phosphogluconate dehydratase (Edd), 2-keto-3-deoxy-6-phosphogluconate aldolase (Eda), phosphoenolpyruvate synthase (PpsA), biotin carboxyl carrier protein (AccB), bifunctional aconitate hydratase 2/2-methylisocitrate dehydratase (AcnB), type II citrate synthase (GltA) and fumarate hydratase (FumA) were enzymes of the core carbon metabolism that were significantly reduced (p-value < 0.05) in *H. pylori* coccoids compared to spirals. On the other hand, phosphoglycerate kinase (Pgk) was significantly elevated (p-value < 0.05) in coccoids. Each bar on the graph represents mean of triplicates and y-axis represents relative abundance (i.e. total sum of the areas under the peaks). Error bar represents standard deviation.

Among those proteins that were significantly less abundant in coccoids when compared to the spirals, 35 were enzymes involved in diverse metabolism pathways (Supplementary Table S1). These include enzymes that are involved in core carbon metabolism (Fig. 2), amino acid metabolism, nucleotide metabolism, lipid metabolism and the metabolism of vitamins and co-factors (Supplementary Table S1). Conversely, the abundances of 13 metabolic enzymes were increased in coccoids (Supplementary Table S2). Based on the comparison of the relative abundance of key metabolic proteins expressed in the two morphologically differentiated forms of *H. pylori*, coccoids were less metabolically active than the spirals but maintained basal levels of these enzymes possibly paving the way for its survival over long period. It is well established that reduced metabolism is a means of survival strategy in living cells^24^. Though low in metabolic activity, these coccoids remain viable^7^, or commonly known as the viable but non-culturable (VBNC). It is therefore poignant to note that the 3-month old cultures did not yield any growth on sub-culturing in fresh liquid or solid media but proteome composition of the coccoid form shows the continuous presence of metabolic proteins, rightfully qualified its VBNC nature.

Endospore forming bacteria maintain longevity by means of forming the metabolically dormant endospores as a survival strategy^25^. Similarly, *H. pylori*, in the form of coccoid, may actively restrict its metabolic activity to basal level in the face of adversity^26^,^27^. As we know, the human stomach is a very dynamic environment with continuous peristalsis and shedding of its overlying mucin layer and gastric epithelial cells^28^. In order to chronically persist in the human stomach^3^,^4^, it is only rational that the bacterium must maintain a basal level of replication and multiplication to sustain its presence in the host stomach. Thus, the coccoid form may be considered as an example of ‘longevity as a solution to adversity’ in the bacterial world. (The phase “longevity as a solution to adversity” is borrowed from the book “Life” of Martha Holmes and Michael Gunton^29^, in which they used to describe the survival of several thousand years old bristlecone pine in the harsh environment in the White Mountains of western USA).

### DNA replication

Whether coccoid *H. pylori* is capable of DNA synthesis and repair remains debatable. Bode *et al*.^16^ had detected newly synthesized DNA among coccoid *H. pylori*, together with polyphosphates that can serve as energy and phosphorus source permitting basal level of endogenous metabolism to preserve the integrity of nucleic acid^16^. Similarly, Hua and Ho (1998) had also revealed that the conservation of DNA composition in *H. pylori* of various ages in the course of morphological changes. On the contrary, Kusters *et al*.^30^ claimed that coccoid were merely morphologic manifestation of bacterial cell death with reduced quantity and integrity DNA and RNA^30^. Our proteome analysis of the coccoids revealed that the abundances of DNA gyrase subunit A (GyrA) and B (GyrB), which are involved in DNA replication in bacteria, were significantly increased as compared to the spirals (Supplementary Table S2). These essential enzymes regulate the conformational changes in DNA topology by catalyzing the concerted breakage and rejoining of DNA strands during normal cellular growth.

Homologous recombination plays important role in maintaining integrity of the genome. Recombinase A (RecA) was elevated in *H. pylori* coccoid (Supplementary Table S2) suggesting that RecA was expressed at high level in order to repair DNA damage or facilitate recombination, which is an important function even in its coccoid form. *H. pylori* RecA has been reported to be highly expressed in both exponential and stationary phase as part of the constitutive DNA damage adaptation system^31^. In addition to the constitutive DNA damage adaptation system, *H. pylori* also relies on other recombinational repair proteins (RecN and RecR) for repairing DNA damage induced by oxidative and acid stresses^32^,^33^. The abundance of RecN and RecR decreased in coccoid compared to spiral (Supplementary Table S1) suggesting that *H. pylori* coccoid may have limited ability to repair DNA damage induced by oxidative and acid stresses. This augurs well with the fact that coccoid maintains low metabolic activity to avoid undue stresses, thus keeping it viable but not culturable. Alternatively, the coccoid form may be less exposed to stress-induced DNA damage and thus the need for RecN and RecR is reduced as compared to its spiral counterpart.

### Environmental adaptation

The abundances of nickel responsive regulator (NikR) and two putative transcriptional regulators were increased in coccoid (Supplementary Table S2). NikR is a nickel-dependent outer membrane protein that regulates the expression of multiple genes by binding to DNA, as well as controlling enzymes that require nickel as co-factor (e.g. urease and hydrogenase) by regulating nickel homeostasis^34^,^35^. Thus, it is not surprising that the abundance of urease accessory protein (UreG), a specific nickel-dependent GTPase^36^, was also increased in coccoid (Supplementary Table S2). However, urease structural subunit B (UreB) and accessory protein (UreF) were reduced in coccoid (Supplementary Table S1), which explained the observation of reduced urease activity of this morphological form.

On the other hand, the abundance of response regulator (CheY) decreased in coccoid (Supplementary Table S1) while that of another response regulator jhp0403 increased in coccoid (Supplementary Table S2). These findings indicate that the coccoid form may possess an active response or ability of adaptation by the bacterium to nutrient depletion stress in a prolonged culture. These CheY response regulators or outer membrane proteins R (OmpR) are essential for chemotaxis and persistent colonization of the gastric mucosa, as well as a part of the stress-responsive operon^37^. These response regulators may also be involved in regulating the physiology of *H. pylori* in response to environmental stimuli and stress, and important in facilitating adaptation to the different environments of the gastric mucosa. CheY has been associated with repression of biofilm formation or dispersion in *Vibrio* species^38^. It is tempting to speculate that CheY may have a significant role in the survival of *H. pylori* in extra-gastric environment.

### Cell wall synthesis and division

**Figure 3:**
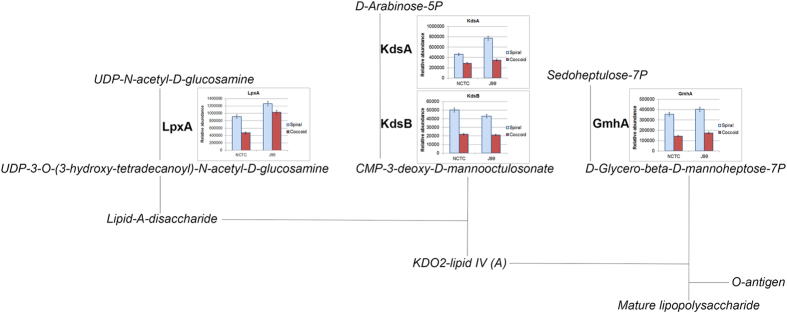
Lipopolysaccharide (LPS) biosynthesis pathway. UPD-N-acetylglucosamine acyltransferase (LpxA), 2-dehydo-3-deoxyphosphooctonate aldolase (KdsA), 3-deoxy-manno-octulosonate cytidylyltransferase (KdsB) and phosphoheptose isomerase (GmnA) were key enzymes involve in lipid A biosynthesis that were significantly reduced (p-value < 0.05) in *H. pylori* coccoids compared to spirals. Each bar on the graph represents mean of triplicates and y-axis represents relative abundance (i.e. total sum of the areas under the peaks). Error bar represents standard deviation.

Lipopolysaccharide (LPS) is a major component of the Gram-negative bacteria cell wall. The expression of UDP-N-acetylglucosamine acyltransferase (LpxA), 2-dehydro-3-deoxyphosphooctonate aldolase (kdsA), 3-deoxy-manno-octulosonate cytidylyltransferase (kdsB) and phosphoheptose isomerase (GmhA) were shown to be lower in coccoid compared to spiral (Supplementary Table S1). These are proteins involved in biosynthesis of LPS lipid A (Fig. 3).

Rod shape-determining protein (MreB) was enhanced in coccoids (Supplementary Table S1). Actin-like MreB is not involved in the maintenance of cell shape, but affects the progression of the cell cycle in *H. pylori* showing that MreB is needed for cell division^39^. The enhancement of MreB suggests it may play a role in the survival of coccoids. In addition, the abundance of cell division inhibitor (MinD) was increased in coccoids (Supplementary Table S2). In *H. pylori*, MinD interacts with MinC to play a role in maintaining proper cell morphology and cell division as *min*C mutant has been demonstrated to form filamentous cells indicating a deficiency in ability to divide^40^.

### Virulence factors

The abundance of tumor necrosis factor-alpha (TNF-alpha)-inducing protein (Hps) was found to be increased in coccoid compared to spiral of *H. pylori* culture (Supplementary Table S2). Hps binds to nucleolin on cell surface and is incorporated into cytosol and nucleus, where it strongly induces expression of TNF-alpha and chemokine genes mediated through NF-kappaB activation, resulting in tumor development^41^,^42^. Hps is an essential factor in *H. pylori* inflammation and cancer microenvironment in the human stomach^43^. Furthermore, Hps could possibly contribute to persistent survival of the bacterium inside the stomach by virtue of its ability to suppress acid production through the induction TNF-alpha and to induce of macrophage apoptosis. The presence of these immune response activators in coccoid further stresses the probable viability of this morphological form of *H. pylori*.

The Sec dependent pathway and type IV secretion system (T4SS) are mechanisms used by bacteria to translocate proteins across their cytoplasmic membrane. *H. pylori* possesses a functional Sec machinery^44^. The abundance of putative inner membrane protein translocase component (YidC) of the Sec-dependent pathway increased in *H. pylori* coccoid (Supplementary Table S2). In addition, the abundances of proteins of the T4SS (CagE and CagV) also increased in coccoid (Supplementary Table S2). CagT, which is an essential structural component of the cag PAI apparatus, acts as a chaperone-like protein to promote the translocation of CagA, a bacterial oncoprotein, into host epithelial cells^45^. Consistent with our finding, antibiotic-induced coccoid forms of two clinical isolates has also been demonstrated to express higher rate of *cagE* mRNA than their spiral counterparts^46^. These results suggest that *H. pylori* coccoid is not a passive entity but may actively infect the human by the expression of various virulence genes over a long period of time in the stomach and probably plays a role in chronic as well as severe gastric diseases.

*H. pylori* coccoid has been observed to be closely associated with severely damaged gastric mucous cells^3^. This suggests that *H. pylori* coccoid can be as virulent, if not more, than the spiral which has been directly or indirectly responsible for greater host cell destruction. Alternatively, the conversion from spiral to coccoid may be the bacterium’s response to host immune system thereby inflicting damage to the gastric mucosa. Our findings are in favor of the former explanation as the abundance of various proteins conferring virulence increased in coccoid when compared to the spiral form, suggesting that *H. pylori* coccoid may be more virulent than spiral in contributing to severe pathogenesis (gastric cancer).

In summary, we have demonstrated that the protein profile of *H. pylori* coccoid from prolonged (3-month old) culture suggests that despite its low metabolic activities, might still preserve its ability to replicate and multiply as coccoid without reverting to spiral form. Most interestingly, these data also indicate that *H. pylori* coccoid may be potentially more virulent than its spiral counterpart. Therefore, it is necessary to further investigate the roles of *H. pylori* coccoid in pathogenesis, as well as in environmental transmission.

## Methods

### *H. pylori* Cultures

*H. pylori* NCTC 11637 (ATCC 43504; American Type Culture Collection, USA) and J99 strains (ATCC 700824) were grown in 100 ml bottles containing 10 ml of brain heart infusion broth (Oxoid, UK) supplemented with 0.4% yeast extract (Oxoid) and 1% β-cyclodextrin (Sigma-Aldrich, USA) in a humidified 10% CO_2_ incubator under stationary conditions. The spiral form was harvested after 3 days while coccoid form was harvested after 3 months. Samples were prepared in independent biological triplicates for protein extraction. Purity of the coccoid culture was confirmed by showing no growth on non-selective chocolate agar supplemented with 5% defibrinated horse blood (HemoStat Laboratories, USA) when incubated for a period of 2 weeks.

### Preparation of Proteome Samples

For the preparation of protein extracts from *H. pylori*, bacteria were harvested by centrifugation (12,000 g, 10 min, 4 °C) from 3-day and 3-month old cultures. The bacterial pellets were washed thrice with sterile Phosphate buffered saline (pH 7.4). The collected cells were lysed and protein extracted using the ProteoSpin detergent-free total protein isolation kit (Norgen Biotek, Canada). Halt protease and phosphatase inhibitors cocktail (Thermo Scientific, USA) were added to the lysate. The lysates were subsequently treated with 10 mM dithiothreitol (DTT; Bio-Rad, USA) at 37 °C for 1 h and alkylated with 55 mM iodoacetamide (IAA; Bio-Rad) for 30 min at room temperature. The proteins in the sample were digested with 1:50 (trypsin: protein) of MS grade porcine trypsin (Calbiochem, Germany) at 37 °C overnight. The samples were desalted using a Pierce C-18 spin column (Thermo Scientific) and dried to completeness in a refrigerated CentriVap centrifugal vacuum concentrator (Labconco, USA) before mass spectrometry analysis. Protein extracts from biological triplicates were pooled for LC-MS/MS analysis.

### LC-MS/MS Analysis

TripleTOF™ 5600 (AB SCIEX, USA) was used for the *H. pylori* spiral and coccoid proteomes as previously described^47^. The instrument was coupled with an Eksigent NanoLC-ultra 2D+ with Nanoflex cHiPLC nanoflex system in Trap-Elute mode for peptide separation. Solvent A contained 0.1% formic acid (v/v) in 2% acetonitrile while solvent B contained 0.1% formic acid (v/v) in 98% acetonitrile. The samples (2 μg each) were loaded onto a cHiPLC trap column (200 μm × 500 μm ChromXP C18-CL, 3 μm, 300 Å) with a flow rate of 3 μL/min for 10 min and separated on a nano cHiPLC analytical column (75 μm × 15 cm ChromXP C18-CL, 3 μm, 300 Å) with a linear gradient of 5 to 40% solvent B for 55 min; 40 to 60% for 8 min; and 60 to 90% for 3 min at a flow rate of 300 nL/min on the Nanoflex cHiPLC system. The Chip nanoLC column was rinsed with 90% solvent B for 5 min and equilibrating with 95% solvent A for 13 min. For data-dependent acquisition experiment, 250-ms survey scan (TOF-MS) and 50-ms automated MS/MS product ion scan for the top 40 ions with the highest intensity was performed. The MS/MS triggering criteria for parent ions were as follows: precursor intensity (>125 counts), charge state (2–5) with dynamic exclusion time of 10 sec and collision energy set as rolling CE script based on m/z and charged state of the precursors. For SWATH MS-based experiments, the mass spectrometer was used in the looped product ion mode with 25 Da mass/windows (each SWATH window has a 1 Da overlap) in the range of 350 to 1,250 Da [(experiment 1: MS1 survey scan (350–1,250 Da); experiment 2: 350–375 Da; experiment 3: 374–400 Da ~ experiment 37: 1,224- 1,250 Da)]. The MS2 scan range was set to 100–1,800 m/z. The collision energy for each SWATH window was 35 V ± 15 V. An accumulation time of 75 ms was used for each SWATH product ion scan and 50 ms for the survey scans (total duty cycle, 2.8 s) in high sensitivity mode. Each sample was analyzed in technical triplicates.

### Data Analysis

All spectra generated from information-dependent acquisitions (IDA) were searched against the *H. pylori* J99 database (1,488 predicted protein entities derived from specific NCBInr database) using ProteinPilot (version 4.5). Peptide library was generated from triplicate runs of IDA analysis for each of the 4 samples (giving a total of 12 runs) and the libraries were combined to create a single common peptide library used for SWATH analysis. The ProteinPilot software generates ion libraries based on FDR of 1%, 5% and 10% automatically during database search^48^. The identified peptides in the search result served as an ion library to apply to the SWATH acquisition runs. For SWATH-MS data, information (including FDR) for the correct peptide peaks extracted (i.e. retention time, amino acid sequence, protein information, etc) compiled from the ProteinPilot ion libraries were processed using the SWATH Acquisition Microapp (version 1.0) in the PeakView software (version 1.2)^22^,^48^. Following the SWATH workflow, the number of proteins first imported into the SWATH analysis software was restricted to the reported protein number based on false discovery rate of 1% and peak mass accuracy of 50 ppm by user input. Peaks were extracted based on software criteria (non-user input), such as mass accuracy (within 50 ppm), charge state, retention time, peak width and area, etc. The peak extraction mass window was 50 ppm and within 4 min of their expected retention time. Peak area for each protein was extracted using 3 peptides (excluding shared) and 3 transitions for all the samples. The peptide list was also further filtered by peptide confidence level (set as 95% based on ProteinPilot scoring definition) and excluding Shared or Modifications. All quantitative analyses (e.g. normalization, statistical calculations) were processed using the MarkerView (version 1.2.1) software. Samples were grouped according to NCTC 11637 spiral, NCTC 11637 coccoid, J99 spiral and J99 coccoid as triplicate data were imported. Normalization was performed based on total sum of the areas under the peaks in the software. Following that, Student’s t-test was performed to compare between these groups, using p-value scoring of 0.05 as cutoff and area fold change as significant expression changes.

## Additional Information

**How to cite this article**: Loke, M. F. *et al*. Understanding the dimorphic lifestyles of human gastric pathogen *Helicobacter pylori* using the SWATH-based proteomics approach. *Sci. Rep.* **6**, 26784; doi: 10.1038/srep26784 (2016).

## Supplementary Material

Supplementary Information

Supplementary Information

Supplementary Information

Supplementary Information

Supplementary Information

Supplementary Information

Supplementary Information

Supplementary Information

Supplementary Information
