# Supplementary material for: Understanding the dimorphic lifestyles of human gastric pathogen Helicobacter pylori using the SWATH-based proteomics approach
Source: Sci Rep. 2016 May 25;6:26784. doi: 10.1038/srep26784 (PMC4879699; doi:10.1038/srep26784)
Supplement: Supplementary Information [file srep26784-s1.doc]

**Understanding the dimorphic lifestyles of human gastric pathogen *Helicobacter pylori* using the SWATH-based proteomics approach**

Mun Fai Loke^1*^, Chow Goon Ng^2^, Yeespana Vilashni^1^, Justin Lim^3^, Bow Ho^2*^

^1^Department of Medical Microbiology, Faculty of Medicine, University of Malaya, Kuala Lumpur, Malaysia

^2^Department of Microbiology, Yong Loo Lin School of Medicine, National University of Singapore, Singapore

^3^AB SCIEX Ltd, Singapore

**Supplementary Table S1: List of proteins showing statistical significance between spiral and coccoid (p<0.05) and fold-change <1.0 (coccoid/ spiral).**

| **Accession** | **Protein** | **Description** | **NCTC 11637** | | | | **J99** | | | |
| --- | --- | --- | --- | --- | --- | --- | --- | --- | --- | --- |
|  |  |  | **Mean intensity (spiral)** | **Mean intensity (coccoid)** | **Fold-change** | **t-test** | **Mean intensity (spiral)** | **Mean intensity (coccoid)** | **Fold-change** | **t-test** |
| **Carbon metabolism** | | | | | | | | | | |
| jhp0111 | PpsA | phosphoenolpyruvate synthase; Catalyzes the phosphorylation of pyruvate to phosphoenolpyruvate (By similarity) | 1198525.3 | 897687.1 | 0.75 | 0.0201 | 1675187.7 | 826199.8 | 0.49 | 0.0000 |
| jhp0022 | GltA | type II citrate synthase | 2366006.4 | 805604.4 | 0.34 | 0.0000 | 2232435.2 | 1751476.9 | 0.78 | 0.0001 |
| jhp0172 |  | hypothetical protein | 1296823.0 | 276373.0 | 0.21 | 0.0009 | 662865.1 | 487640.3 | 0.74 | 0.0316 |
| jhp1010 | AccB | biotin carboxyl carrier protein | 515861.1 | 186580.6 | 0.36 | 0.0008 | 397584.1 | 220446.9 | 0.55 | 0.0006 |
| jhp1029 | Glk | Glucokinase | 383821.8 | 109868.4 | 0.29 | 0.0010 | 369943.4 | 192858.8 | 0.52 | 0.0000 |
| jhp0716 | AcnB | bifunctional aconitate hydratase 2/2-methylisocitrate dehydratase | 2059292.2 | 951387.1 | 0.46 | 0.0009 | 3330491.6 | 845438.9 | 0.25 | 0.0000 |
| jhp0252 | Orf1 | chlorohydrolase | 1524763.4 | 173658.2 | 0.11 | 0.0008 | 782346.2 | 468120.1 | 0.60 | 0.0001 |
| jhp0162 | Fba | fructose-bisphosphate aldolase; Catalyzes the aldol condensation of dihydroxyacetone phosphate (DHAP or glycerone-phosphate) with glyceraldehyde 3- phosphate (G3P) to form fructose 1,6-bisphosphate (FBP) in gluconeogenesis and the reverse reaction in glycolysis (By similarity) | 4505312.5 | 1026231.6 | 0.23 | 0.0004 | 2592883.6 | 848449.3 | 0.33 | 0.0001 |
| jhp1440 | Fbp | fructose-1,6-bisphosphatase | 2443382.6 | 415189.2 | 0.17 | 0.0001 | 640106.9 | 346537.2 | 0.54 | 0.0205 |
| jhp0638 | Thl | acetyl-COA acetyltransferase | 353762.5 | 137976.7 | 0.39 | 0.0058 | 300204.4 | 87204.5 | 0.29 | 0.0000 |
| jhp1025 | Eda | 2-keto-3-deoxy-6-phosphogluconate aldolase | 1820376.2 | 164632.3 | 0.09 | 0.0000 | 549999.3 | 260697.4 | 0.47 | 0.0004 |
| jhp1026 | Edd | phosphogluconate dehydratase | 820522.0 | 217484.4 | 0.27 | 0.0000 | 496353.1 | 183427.5 | 0.37 | 0.0011 |
| jhp0171 | GlyA | serine hydroxymethyltransferase; Interconversion of serine and glycine | 445994.0 | 73536.9 | 0.16 | 0.0017 | 414983.8 | 192595.9 | 0.46 | 0.0030 |
| jhp0023 | Icd | isocitrate dehydrogenase | 8672368.7 | 909906.5 | 0.10 | 0.0083 | 8450438.8 | 2791922.7 | 0.33 | 0.0002 |
| jhp1245 | FumA | fumarate hydratase | 2050958.5 | 167759.4 | 0.08 | 0.0002 | 992914.8 | 306406.3 | 0.31 | 0.0001 |
| **Amino acid metabolism** | | | | | | | | | | |
| jhp0517 | PepA | leucyl aminopeptidase; Presumably involved in the processing and regular turnover of intracellular proteins. Catalyzes the removal of unsubstituted N-terminal amino acids from various peptides (By similarity) | 9851927.3 | 4318348.1 | 0.44 | 0.0001 | 6711100.9 | 6294303.8 | 0.94 | 0.0259 |
| jhp1011 | AccC | biotin carboxylase | 670625.9 | 379053.8 | 0.57 | 0.0001 | 1024422.4 | 515039.4 | 0.50 | 0.0129 |
| jhp0984 | SerA | D-3-phosphoglycerate dehydrogenase | 778391.7 | 422284.8 | 0.54 | 0.0002 | 1611958.8 | 716566.8 | 0.44 | 0.0001 |
| jhp0279 | AmiE | acylamide amidohydrolase; Catalyzes the hydrolysis of short-chain aliphatic amides to their corresponding organic acids with release of ammonia. Hydrolyzes propionamide, acetamide and acrylamide, but has no activity with formamide or urea. The natural substrates of amiE in its gastric environment are not known. Probably functions to ensure nitrogen supply to the bacteria | 3941029.9 | 974144.1 | 0.25 | 0.0002 | 2492889.5 | 1590770.1 | 0.64 | 0.0004 |
| jhp0615 | AspB | aspartate aminotransferase | 1066048.9 | 243104.1 | 0.23 | 0.0010 | 754732.0 | 493850.8 | 0.65 | 0.0021 |
| jhp0594 | AspA | aspartate ammonia-lyase | 1386753.3 | 236053.9 | 0.17 | 0.0006 | 1089253.6 | 608433.6 | 0.56 | 0.0018 |
| jhp0962 | SpeA | arginine decarboxylase | 540946.1 | 126215.7 | 0.23 | 0.0018 | 284866.7 | 172961.4 | 0.61 | 0.0105 |
| jhp0673 |  | putative aminotransferase | 152450.8 | 59028.3 | 0.39 | 0.0055 | 97128.7 | 33393.0 | 0.34 | 0.0184 |
| jhp0386 | AroQ | 3-dehydroquinate dehydratase; Catalyzes a trans-dehydration via an enolate intermediate (By similarity) | 3111342.5 | 385440.1 | 0.12 | 0.0003 | 1783320.9 | 944754.9 | 0.53 | 0.0004 |
| **Nucleotide metabolism** | | | | | | | | | | |
| jhp0519 | Apt | adenine phosphoribosyltransferase; Catalyzes a salvage reaction resulting in the formation of AMP, that is energically less costly than de novo synthesis | 1672476.7 | 1478746.1 | 0.88 | 0.0147 | 507994.4 | 470692.2 | 0.93 | 0.0310 |
| jhp0768 | GuaB | inosine 5'-monophosphate dehydrogenase | 955161.5 | 603699.1 | 0.63 | 0.0004 | 1057279.7 | 621322.2 | 0.59 | 0.0007 |
| jhp0710 |  | hypothetical protein | 354869.2 | 209377.4 | 0.59 | 0.0332 | 536835.6 | 288027.5 | 0.54 | 0.0146 |
| jhp1009 | Dcd | deoxycytidine triphosphate deaminase | 565754.6 | 286780.2 | 0.51 | 0.0014 | 441275.8 | 247998.6 | 0.56 | 0.0021 |
| jhp0607 | Rnc | ribonuclease III; Digests double-stranded RNA. Involved in the processing of ribosomal RNA precursors and of some mRNAs (By similarity) | 55394.9 | 15761.2 | 0.28 | 0.0036 | 92405.2 | 60303.7 | 0.65 | 0.0156 |
| jhp0713 | RpoZ | DNA-directed RNA polymerase subunit omega; Promotes RNA polymerase assembly. Latches the N- and C- terminal regions of the beta' subunit thereby facilitating its interaction with the beta and alpha subunits (By similarity) | 30175.7 | 12239.0 | 0.41 | 0.0309 | 36762.0 | 13145.6 | 0.36 | 0.0460 |
| **Lipid metabolism** | | | | | | | | | | |
| jhp0805 | Cdh | CDP-diacylglycerol pyrophosphatase | 41675.6 | 15133.3 | 0.36 | 0.0315 | 28267.4 | 12698.5 | 0.45 | 0.0275 |
| **Metabolism of vitamins and co-factors** | | | | | | | | | | |
| jhp0222 | HemC | porphobilinogen deaminase; Tetrapolymerization of the monopyrrole PBG into the hydroxymethylbilane pre-uroporphyrinogen in several discrete steps | 187611.5 | 84498.8 | 0.45 | 0.0349 | 264673.0 | 129759.7 | 0.49 | 0.0020 |
| jhp0610 | HemN | coproporphyrinogen III oxidase; Anaerobic transformation of coproporphyrinogen-III into protoporphyrinogen-IX (By similarity) | 429081.0 | 312767.8 | 0.73 | 0.0211 | 712778.1 | 543217.6 | 0.76 | 0.0040 |
| jhp0291 | HemL | glutamate-1-semialdehyde aminotransferase | 224150.4 | 149592.4 | 0.67 | 0.0269 | 461236.5 | 272647.2 | 0.59 | 0.0045 |
| jhp1489 | PdxJ | pyridoxine 5'-phosphate synthase; Catalyzes the complicated ring closure reaction between the two acyclic compounds 1-deoxy-D-xylulose-5-phosphate (DXP) and 3-amino-2-oxopropyl phosphate (1-amino-acetone-3-phosphate or AAP) to form pyridoxine 5'-phosphate (PNP) and inorganic phosphate (By similarity) | 1637700.0 | 351748.5 | 0.21 | 0.0009 | 1721931.4 | 1468743.1 | 0.85 | 0.0010 |
| **Lipopolysaccharide biosynthesis** | | | | | | | | | | |
| jhp1289 | LpxA | UDP-N-acetylglucosamine acyltransferase; Involved in the biosynthesis of lipid A, a phosphorylated glycolipid that anchors the lipopolysaccharide to the outer membrane of the cell (By similarity) | 1035753.2 | 544539.8 | 0.53 | 0.0023 | 1475692.5 | 1177441.6 | 0.80 | 0.0060 |
| jhp0003 | KdsA | 2-dehydro-3-deoxyphosphooctonate aldolase | 531792.8 | 329989.4 | 0.62 | 0.0020 | 911382.9 | 412258.9 | 0.45 | 0.0001 |
| jhp0215 | KdsB | 3-deoxy-manno-octulosonate cytidylyltransferase; Activates KDO (a required 8-carbon sugar) for incorporation into bacterial lipopolysaccharide in Gram-negative bacteria (By similarity) | 57860.1 | 25403.8 | 0.44 | 0.0409 | 50843.8 | 25007.3 | 0.49 | 0.0058 |
| jhp0791 | GmhA | phosphoheptose isomerase; Catalyzes the isomerization of sedoheptulose 7-phosphate in D-glycero-D-manno-heptose 7-phosphate (By similarity) | 408931.6 | 162979.2 | 0.40 | 0.0052 | 475622.7 | 221304.0 | 0.47 | 0.0007 |
| **Replication and repair** | | | | | | | | | | |
| jhp1066 | ParB | plasmid replication-partition related protein; Involved in chromosome partition. Localize to both poles of the predivisional cell following completion of DNA replication. Binds to the DNA origin of replication (By similarity) | 603606.2 | 363290.7 | 0.60 | 0.0311 | 709899.9 | 526497.9 | 0.74 | 0.0005 |
| jhp1434 | RecN | DNA repair protein(recombination protein N); May be involved in recombinational repair of damaged DNA (By similarity) | 31906.9 | 10604.9 | 0.33 | 0.0325 | 36137.1 | 20996.9 | 0.58 | 0.0183 |
| jhp0859 | RecR | recombination protein RecR; May play a role in DNA repair. It seems to be involved in an recBC-independent recombinational process of DNA repair. It may act with recF and recO (By similarity) | 41321.6 | 17097.3 | 0.41 | 0.0049 | 22468.5 | 10980.9 | 0.49 | 0.0356 |
| **RNA degradation** | | | | | | | | | | |
| jhp0232 | DeaD | ATP-dependent RNA helicase DeaD | 452758.7 | 362935.6 | 0.80 | 0.0420 | 668542.8 | 322373.6 | 0.48 | 0.0004 |
| **Protein folding, sorting and degradation** | | | | | | | | | | |
| jhp0102 | GrpE | 24kDa chaperone; Participates actively in the response to hyperosmotic and heat shock by preventing the aggregation of stress-denatured proteins, in association with dnaK and grpE. It is the nucleotide exchange factor for dnaK and may function as a thermosensor. Unfolded proteins bind initially to dnaJ; upon interaction with the dnaJ-bound protein, dnaK hydrolyzes its bound ATP, resulting in the formation of a stable complex. GrpE releases ADP from dnaK; ATP binding to dnaK triggers the release of the substrate protein, thus completing the reaction cycle. Several rounds of ATP- depende [...] | 631860.3 | 276345.8 | 0.44 | 0.0009 | 595153.7 | 408768.3 | 0.69 | 0.0024 |
| jhp0249 | ClpB | heat shock protein; Part of a stress-induced multi-chaperone system, it is involved in the recovery of the cell from heat-induced damage, in cooperation with dnaK, dnaJ and grpE. Acts before dnaK, in the processing of protein aggregates. Protein binding stimulates the ATPase activity; ATP hydrolysis unfolds the denatured protein aggregates, which probably helps expose new hydrophobic binding sites on the surface of clpB-bound aggregates, contributing to the solubilization and refolding of denatured protein aggregates by dnaK (By similarity). Necessary for surviving high-temperature stress | 1259331.2 | 361204.6 | 0.29 | 0.0002 | 1000598.4 | 619452.5 | 0.62 | 0.0197 |
| jhp0730 | ClpP | ATP-dependent Clp protease proteolytic subunit; Cleaves peptides in various proteins in a process that requires ATP hydrolysis. Has a chymotrypsin-like activity. Plays a major role in the degradation of misfolded proteins (By similarity) | 90621.6 | 39836.7 | 0.44 | 0.0012 | 121687.7 | 27162.4 | 0.22 | 0.0007 |
| **Translation** | | | | | | | | | | |
| jhp1444 | Tsf | elongation factor Ts; Associates with the EF-Tu.GDP complex and induces the exchange of GDP to GTP. It remains bound to the aminoacyl-tRNA.EF- Tu.GTP complex up to the GTP hydrolysis stage on the ribosome | 2724576.5 | 1655022.3 | 0.61 | 0.0053 | 2914484.4 | 2491285.7 | 0.85 | 0.0019 |
| jhp0156 |  | hypothetical protein | 570829.4 | 285548.7 | 0.50 | 0.0245 | 318787.6 | 217155.7 | 0.68 | 0.0028 |
| jhp1118 | Efg | elongation factor G; This protein promotes the GTP-dependent translocation of the nascent protein chain from the A-site to the P-site of the ribosome | 4806336.3 | 2497610.6 | 0.52 | 0.0014 | 5446205.4 | 3184845.7 | 0.58 | 0.0007 |
| jhp1373 | SerS | seryl-tRNA synthetase; Catalyzes the attachment of serine to tRNA(Ser). Is also able to aminoacylate tRNA(Sec) with serine, to form the misacylated tRNA L-seryl-tRNA(Sec), which will be further converted into selenocysteinyl-tRNA(Sec) (By similarity) | 253728.6 | 175572.6 | 0.69 | 0.0043 | 453031.7 | 177900.2 | 0.39 | 0.0084 |
| jhp0978 | PheS | phenylalanyl-tRNA synthetase subunit alpha | 324941.8 | 190453.4 | 0.59 | 0.0062 | 541725.6 | 242803.0 | 0.45 | 0.0108 |
| jhp1075 | TrmD | tRNA (guanine-N(1)-)-methyltransferase; Specifically methylates guanosine-37 in various tRNAs (By similarity) | 59541.9 | 21532.1 | 0.36 | 0.0015 | 46801.0 | 31071.1 | 0.66 | 0.0160 |
| jhp0769 | GatA | aspartyl/glutamyl-tRNA amidotransferase subunit A; Furnishes a means for formation of correctly charged Gln-tRNA(Gln) through the transamidation of misacylated Glu- tRNA(Gln) in organisms which lack glutaminyl-tRNA synthetase. The reaction takes place in the presence of glutamine and ATP through an activated gamma-phospho-Glu-tRNA(Gln) (By similarity) | 690203.7 | 308131.5 | 0.45 | 0.0048 | 426430.4 | 202057.6 | 0.47 | 0.0001 |
| jhp0603 | GatB | aspartyl/glutamyl-tRNA amidotransferase subunit B; Allows the formation of correctly charged Asn-tRNA(Asn) or Gln-tRNA(Gln) through the transamidation of misacylated Asp- tRNA(Asn) or Glu-tRNA(Gln) in organisms which lack either or both of asparaginyl-tRNA or glutaminyl-tRNA synthetases. The reaction takes place in the presence of glutamine and ATP through an activated phospho-Asp-tRNA(Asn) or phospho-Glu-tRNA(Gln) (By similarity) | 814950.2 | 287448.1 | 0.35 | 0.0012 | 578743.0 | 364557.8 | 0.63 | 0.0029 |
| jhp0979 | PheT | phenylalanyl-tRNA synthetase subunit beta | 628284.1 | 175903.4 | 0.28 | 0.0004 | 677050.7 | 453638.7 | 0.67 | 0.0038 |
| jhp0432 | TypA | hypothetical protein; Not known; probably interacts with the ribosomes in a GTP dependent manner (By similarity) | 2000155.0 | 850165.0 | 0.43 | 0.0008 | 2652990.2 | 1351354.7 | 0.51 | 0.0001 |
| jhp1177 | Frr | ribosome recycling factor; Responsible for the release of ribosomes from messenger RNA at the termination of protein biosynthesis. May increase the efficiency of translation by recycling ribosomes from one round of translation to another (By similarity) | 63079.2 | 35837.6 | 0.57 | 0.0020 | 181487.1 | 61812.4 | 0.34 | 0.0002 |
| **Ribosome** | | | | | | | | | | |
| jhp1212 | RplQ | 50S ribosomal protein L17 | 276125.0 | 178372.7 | 0.65 | 0.0284 | 282025.3 | 208111.5 | 0.74 | 0.0095 |
| jhp1238 | RplD | 50S ribosomal protein L4; One of the primary rRNA binding proteins, this protein initially binds near the 5'-end of the 23S rRNA. It is important during the early stages of 50S assembly. It makes multiple contacts with different domains of the 23S rRNA in the assembled 50S subunit and ribosome (By similarity) | 1027322.5 | 482597.4 | 0.47 | 0.0110 | 1480419.8 | 689874.3 | 0.47 | 0.0002 |
| jhp1221 | RplO | 50S ribosomal protein L15; Binds to the 23S rRNA (By similarity) | 138705.2 | 76345.4 | 0.55 | 0.0398 | 467254.3 | 116533.6 | 0.25 | 0.0000 |
| **Signal transduction** | | | | | | | | | | |
| jhp1460 | PetB | ubiquinol cytochrome c oxidoreductase, cytochrome b subunit; Component of the ubiquinol-cytochrome c reductase complex (complex III or cytochrome b-c1 complex), which is a respiratory chain that generates an electrochemical potential coupled to ATP synthesis (By similarity) | 169514.0 | 67601.0 | 0.40 | 0.0001 | 144879.8 | 98302.1 | 0.68 | 0.0001 |
| jhp0546 |  | methyl-accepting chemotaxis protein (MCP) | 1212855.8 | 617601.4 | 0.51 | 0.0000 | 1286149.8 | 688048.6 | 0.53 | 0.0000 |
| jhp0461 | GlnA | glutamine synthetase | 1084821.9 | 382754.2 | 0.35 | 0.0005 | 1173493.2 | 797048.3 | 0.68 | 0.0067 |
| jhp0358 | CheY | response regulator; Involved in the transmission of sensory signals from the chemoreceptors to the flagellar motors. CheY seems to regulate the clockwise (CW) rotation (By similarity) | 215360.9 | 107041.8 | 0.50 | 0.0038 | 593167.1 | 241967.7 | 0.41 | 0.0006 |
| **Virulence factor** | | | | | | | | | | |
| jhp0067 | UreB | urease subunit beta; Ammonia produced by ureolysis increases the gastric pH thereby providing an environment permissive for colonization of the stomach (By similarity) | 10054454.5 | 7537067.1 | 0.75 | 0.0014 | 6696458.5 | 4725605.2 | 0.71 | 0.0009 |
| jhp0064 | UreF | urease accessory protein; Required for maturation of urease via the functional incorporation of the urease nickel metallocenter (By similarity) | 127970.2 | 74027.4 | 0.58 | 0.0331 | 177189.6 | 110689.9 | 0.62 | 0.0038 |
| jhp0228 | Dps | neutrophil-activating protein A; Protects DNA from oxidative damage by sequestering intracellular Fe(2+) ion and storing it in the form of Fe(3+) oxyhydroxide mineral. One hydrogen peroxide oxidizes two Fe(2+) ions, which prevents hydroxyl radical production by the Fenton reaction. Required for the survival in the presence of oxidative stress. Dps is also a virulence factor that activates neutrophils, mast cells and monocytes. It binds to neutrophil- glycosphingolipids and to sulfated carbohydrates on mucin. It might have a role in the accumulation of neutrophils and monocytes at the s [...] | 1157974.3 | 756412.5 | 0.65 | 0.0170 | 3241323.9 | 1865194.5 | 0.58 | 0.0296 |
| jhp0992 | Sod | iron-dependent superoxide dismutase; Destroys radicals which are normally produced within the cells and which are toxic to biological systems (By similarity) | 855986.1 | 128497.7 | 0.15 | 0.0000 | 534738.1 | 404181.7 | 0.76 | 0.0002 |
| **Outer membrane protein** | | | | | | | | | | |
| jhp1103 |  | putative outer membrane protein | 1370525.5 | 725397.0 | 0.53 | 0.0367 | 1865091.3 | 1565904.1 | 0.84 | 0.0000 |
| jhp0021 | HopD | putative Outer membrane protein | 1778572.0 | 1571859.2 | 0.88 | 0.0435 | 331280.7 | 138948.6 | 0.42 | 0.0023 |
| jhp1008 |  | putative outer membrane protein | 27246.5 | 6465.1 | 0.24 | 0.0000 | 35950.3 | 14203.2 | 0.40 | 0.0340 |
| **Hypothetical protein** | | | | | | | | | | |
| jhp1144 |  | hypothetical protein | 212675.2 | 150159.7 | 0.71 | 0.0172 | 279865.6 | 172657.9 | 0.62 | 0.0087 |
| jhp0119 |  | hypothetical protein | 832652.1 | 309994.7 | 0.37 | 0.0066 | 465432.7 | 394981.9 | 0.85 | 0.0100 |
| jhp0346 |  | hypothetical protein | 198173.3 | 73632.1 | 0.37 | 0.0115 | 163689.2 | 136478.8 | 0.83 | 0.0072 |
| jhp1318 |  | hypothetical protein | 55292.9 | 35377.5 | 0.64 | 0.0251 | 49769.5 | 25142.7 | 0.51 | 0.0006 |
| jhp0301 |  | hypothetical protein | 2101935.3 | 803593.5 | 0.38 | 0.0005 | 7577499.4 | 4179649.1 | 0.55 | 0.0000 |
| jhp0694 |  | hypothetical protein | 276491.1 | 119653.7 | 0.43 | 0.0072 | 214452.1 | 102683.8 | 0.48 | 0.0011 |
| jhp1494 |  | hypothetical protein | 10716580.7 | 2532593.8 | 0.24 | 0.0033 | 6878177.3 | 4370909.5 | 0.64 | 0.0000 |
| jhp0628 |  | hypothetical protein | 1262579.1 | 228540.7 | 0.18 | 0.0000 | 408436.9 | 256769.5 | 0.63 | 0.0069 |
| jhp0457 |  | hypothetical protein | 174297.2 | 15832.4 | 0.09 | 0.0012 | 63821.4 | 45180.5 | 0.71 | 0.0158 |
| jhp1418 |  | hypothetical protein | 158744.9 | 21211.4 | 0.13 | 0.0001 | 158958.1 | 76662.3 | 0.48 | 0.0004 |

**Supplementary Table S2: List of proteins showing statistical significance between spiral and coccoid (p<0.05) and fold-change >1.0 (coccoid/ spiral).**

| **Accession** | **Protein** | **Description** | **NCTC 11637** | | | | **J99** | | | |
| --- | --- | --- | --- | --- | --- | --- | --- | --- | --- | --- |
|  |  |  | **Mean intensity (spiral)** | **Mean intensity (coccoid)** | **Fold-change** | **t-test** | **Mean intensity (spiral)** | **Mean intensity (coccoid)** | **Fold-change** | **t-test** |
| **Carbon metabolism** | | | | | | | | | | |
| jhp0310 | FlaG | putative flagellar biosynthesis protein | 11509.1 | 33413.0 | 2.90 | 0.0038 | 14438.2 | 29418.2 | 2.04 | 0.0004 |
| jhp0039 | NolK | putative sugar nucleotide biosynthesis | 216736.0 | 400287.0 | 1.85 | 0.0111 | 310497.7 | 536134.2 | 1.73 | 0.0003 |
| jhp0459 | GlcD | putative glycolate oxidase | 182730.5 | 332723.7 | 1.82 | 0.0192 | 295585.3 | 445615.4 | 1.51 | 0.0270 |
| jhp0524 | FolD | methylenetetrahydrofolate dehydrogenase/ methenyltetrahydrofolate cyclohydrolase; Catalyzes the oxidation of 5,10- methylenetetrahydrofolate to 5,10-methenyltetrahydrofolate and then the hydrolysis of 5,10-methenyltetrahydrofolate to 10- formyltetrahydrofolate (By similarity) | 290111.5 | 485304.2 | 1.67 | 0.0092 | 252617.6 | 276110.8 | 1.09 | 0.0045 |
| jhp1264 | Pgk | phosphoglycerate kinase | 1695259.9 | 2739258.8 | 1.62 | 0.0001 | 1972772.8 | 2173363.8 | 1.10 | 0.0327 |
| **Amino acid metabolism** | | | | | | | | | | |
| jhp0207 |  | NifU-like protein | 1884691.8 | 6881135.2 | 3.65 | 0.0000 | 2088439.5 | 4880506.3 | 2.34 | 0.0001 |
| jhp0161 |  | putative peptidyl-prolyl cis-trans isomerase | 225450.3 | 691656.0 | 3.07 | 0.0051 | 330823.1 | 568622.2 | 1.72 | 0.0121 |
| jhp0516 |  | GTP-dependent nucleic acid-binding protein EngD | 523892.3 | 1873106.0 | 3.58 | 0.0001 | 1124592.9 | 1307178.9 | 1.16 | 0.0141 |
| jhp0018 | NspC | putative carboxynorspermidine decarboxylase | 198072.5 | 271690.3 | 1.37 | 0.0384 | 236718.7 | 360713.2 | 1.52 | 0.0056 |
| jhp1361 | IlvE | branched-chain amino acid aminotransferase; Acts on leucine, isoleucine and valine (By similarity) | 427704.4 | 661450.3 | 1.55 | 0.0019 | 358231.5 | 480940.9 | 1.34 | 0.0005 |
| **Nucleotide metabolism** | | | | | | | | | | |
| jhp1158 | CarA | carbamoyl phosphate synthase small subunit | 415751.7 | 1027695.7 | 2.47 | 0.0010 | 504127.8 | 627570.2 | 1.24 | 0.0185 |
| jhp0005 | PyrF | orotidine 5'-phosphate decarboxylase; Catalyzes the decarboxylation of orotidine 5'- monophosphate (OMP) to uridine 5'-monophosphate (UMP) (By similarity) | 217922.9 | 337401.4 | 1.55 | 0.0355 | 183701.0 | 235738.3 | 1.28 | 0.0083 |
| **Metabolism of terpenoids and polyketides** | | | | | | | | | | |
| jhp0981 | IspH | 4-hydroxy-3-methylbut-2-enyl diphosphate reductase; Converts 1-hydroxy-2-methyl-2-(E)-butenyl 4-diphosphate into isopentenyl diphosphate (IPP) and dimethylallyl diphosphate (DMAPP) (By similarity) | 409129.8 | 614803.2 | 1.50 | 0.0005 | 355372.1 | 608266.7 | 1.71 | 0.0002 |
| **Lipopolysaccharide biosynthesis** | | | | | | | | | | |
| jhp0963 |  | putative polysaccharide biosynthesis protein | 219752.3 | 733122.2 | 3.34 | 0.0000 | 362923.8 | 451307.3 | 1.24 | 0.0142 |
| **Replication and repair** | | | | | | | | | | |
| jhp0786 | HsdM_2 | Type I restriction enzyme modification subunit | 74532.9 | 261823.0 | 3.51 | 0.0005 | 240634.2 | 328895.0 | 1.37 | 0.0033 |
| jhp1353 | DnaE | DNA polymerase III subunit alpha; DNA polymerase III is a complex, multichain enzyme responsible for most of the replicative synthesis in bacteria. This DNA polymerase also exhibits 3' to 5' exonuclease activity. The alpha chain is the DNA polymerase (By similarity) | 201658.2 | 429335.7 | 2.13 | 0.0376 | 163175.8 | 201452.7 | 1.23 | 0.0087 |
| jhp0641 | GyrA | DNA gyrase subunit A; DNA gyrase negatively supercoils closed circular double- stranded DNA in an ATP-dependent manner and also catalyzes the interconversion of other topological isomers of double-stranded DNA rings, including catenanes and knotted rings | 827801.4 | 1377079.0 | 1.66 | 0.0000 | 828388.7 | 1329120.5 | 1.60 | 0.0007 |
| jhp0453 | GyrB | DNA gyrase subunit B | 506959.2 | 635001.5 | 1.25 | 0.0248 | 559623.1 | 798062.4 | 1.43 | 0.0002 |
| jhp0152 |  | putative transcriptional regulator | 2116030.2 | 3151545.2 | 1.49 | 0.0122 | 1219485.4 | 2048433.8 | 1.68 | 0.0016 |
| jhp0643 |  | putative transcriptional regulator | 250795.9 | 416209.7 | 1.66 | 0.0007 | 549701.8 | 805392.8 | 1.47 | 0.0000 |
| jhp1257 | NikR | nickel responsive regulator; Transcriptional regulator (Potential) | 441732.2 | 759264.6 | 1.72 | 0.0009 | 315543.3 | 552730.5 | 1.75 | 0.0103 |
| jhp0141 | RecA | recombinase A; Can catalyze the hydrolysis of ATP in the presence of single-stranded DNA, the ATP-dependent uptake of single-stranded DNA by duplex DNA, and the ATP-dependent hybridization of homologous single-stranded DNAs. It interacts with lexA causing its activation and leading to its autocatalytic cleavage (By similarity) | 1085238.8 | 1993339.5 | 1.84 | 0.0011 | 1475565.0 | 1774734.4 | 1.20 | 0.0084 |
| **RNA degradation** | | | | | | | | | | |
| jhp0497 | Rho | transcription termination factor Rho; Facilitates transcription termination by a mechanism that involves Rho binding to the nascent RNA, activation of Rho's RNA-dependent ATPase activity, and release of the mRNA from the DNA template (By similarity) | 475775.9 | 737245.0 | 1.55 | 0.0014 | 861531.7 | 938313.0 | 1.09 | 0.0091 |
| **Protein folding, sorting and degradation** | | | | | | | | | | |
| jhp0009 | GroS | co-chaperonin GroES; Binds to Cpn60 in the presence of Mg-ATP and suppresses the ATPase activity of the latter (By similarity) | 3354437.6 | 12429132.2 | 3.71 | 0.0001 | 3143921.0 | 5157321.1 | 1.64 | 0.0000 |
| jhp1288 | ClpX | ATP-dependent protease ATP-binding subunit ClpX; ATP-dependent specificity component of the Clp protease. It directs the protease to specific substrates. Can perform chaperone functions in the absence of clpP (By similarity) | 322591.6 | 1221838.9 | 3.79 | 0.0000 | 809866.3 | 924676.1 | 1.14 | 0.0012 |
| jhp1269 | Prc | carboxyl-terminal protease | 310053.3 | 508612.2 | 1.64 | 0.0055 | 339799.1 | 751786.0 | 2.21 | 0.0008 |
| jhp0356 | FtsH | ATP-dependent zinc metallopeptidase; Seems to act as an ATP-dependent zinc metallopeptidase (By similarity) | 946963.1 | 1618430.2 | 1.71 | 0.0005 | 1104148.5 | 2120744.1 | 1.92 | 0.0007 |
| **Cell division** | | | | | | | | | | |
| jhp0314 | MinD | cell division inhibitor; ATPase required for the correct placement of the division site. Cell division inhibitors minC and minD act in concert to form an inhibitor capable of blocking formation of the polar Z ring septums. Rapidly oscillates between the poles of the cell to destabilize ftsZ filaments that have formed before they mature into polar Z rings (By similarity) | 781220.8 | 2164010.9 | 2.77 | 0.0003 | 1361518.6 | 1820672.1 | 1.34 | 0.0005 |
| jhp1287 | MreB | rod shape-determining protein MreB | 1043521.5 | 1634038.4 | 1.57 | 0.0017 | 1586950.2 | 2867937.7 | 1.81 | 0.0016 |
| **Translation** | | | | | | | | | | |
| jhp0157 | PrfB | peptide chain release factor 2; Peptide chain release factor 2 directs the termination of translation in response to the peptide chain termination codons UGA and UAA (By similarity) | 177316.3 | 430032.0 | 2.43 | 0.0003 | 204848.7 | 297834.8 | 1.45 | 0.0028 |
| jhp0560 | AspS | aspartyl-tRNA synthetase | 600917.7 | 1545411.3 | 2.57 | 0.0000 | 1079983.1 | 1220086.2 | 1.13 | 0.0037 |
| jhp0501 |  | hypothetical protein | 97691.1 | 167888.0 | 1.72 | 0.0260 | 284618.8 | 374186.5 | 1.31 | 0.0055 |
| jhp0377 | InfB | translation initiation factor IF-2; One of the essential components for the initiation of protein synthesis. Protects formylmethionyl-tRNA from spontaneous hydrolysis and promotes its binding to the 30S ribosomal subunits. Also involved in the hydrolysis of GTP during the formation of the 70S ribosomal complex (By similarity) | 587846.2 | 741639.1 | 1.26 | 0.0168 | 745017.4 | 837433.2 | 1.12 | 0.0383 |
| **Ribosome** | | | | | | | | | | |
| jhp0982 | RpsA | 30S ribosomal protein S1; Binds mRNA; thus facilitating recognition of the initiation point. It is needed to translate mRNA with a short Shine-Dalgarno (SD) purine-rich sequence (By similarity) | 1371168.4 | 5945830.1 | 4.34 | 0.0001 | 1371893.9 | 3161116.0 | 2.30 | 0.0001 |
| jhp1445 | RpsB | 30S ribosomal protein S2 | 1014984.2 | 1612606.2 | 1.59 | 0.0001 | 1443277.8 | 1558543.1 | 1.08 | 0.0272 |
| jhp1215 | RpsK | 30S ribosomal protein S11; Located on the platform of the 30S subunit, it bridges several disparate RNA helices of the 16S rRNA. Forms part of the Shine-Dalgarno cleft in the 70S ribosome (By similarity) | 455068.3 | 888655.8 | 1.95 | 0.0000 | 663795.9 | 882613.1 | 1.33 | 0.0001 |
| jhp0498 | RpmE | 50S ribosomal protein L31; Binds the 23S rRNA (By similarity) | 152929.2 | 257816.4 | 1.69 | 0.0158 | 256461.2 | 325519.3 | 1.27 | 0.0014 |
| jhp0281 | RplU | 50S ribosomal protein L21; This protein binds to 23S rRNA in the presence of protein L20 (By similarity) | 824014.4 | 1309553.8 | 1.59 | 0.0031 | 1007983.7 | 1268640.3 | 1.26 | 0.0398 |
| **Signal transduction** | | | | | | | | | | |
| jhp1062 | AtpA | F0F1 ATP synthase subunit alpha; Produces ATP from ADP in the presence of a proton gradient across the membrane. The alpha chain is a regulatory subunit (By similarity) | 6360777.3 | 17027377.9 | 2.68 | 0.0001 | 6830216.7 | 15241915.0 | 2.23 | 0.0000 |
| jhp1254 | MnmA | tRNA-specific 2-thiouridylase MnmA; Catalyzes the 2-thiolation of uridine at the wobble position (U34) of tRNA, leading to the formation of s(2)U34 (By similarity) | 773551.7 | 1144584.4 | 1.48 | 0.0006 | 338114.6 | 848765.3 | 2.51 | 0.0001 |
| jhp0403 |  | response regulator | 438685.3 | 1004869.0 | 2.29 | 0.0000 | 751828.2 | 1123351.1 | 1.49 | 0.0002 |
| **Virulence factor** | | | | | | | | | | |
| jhp0543 | Hps | tumor necrosis factor alpha-inducing protein | 357080.4 | 574442.1 | 1.61 | 0.0030 | 56508.7 | 173810.1 | 3.08 | 0.0088 |
| jhp0063 | UreG | urease accessory protein; Facilitates the functional incorporation of the urease nickel metallocenter. This process requires GTP hydrolysis, probably effectuated by ureG (By similarity) | 1641944.5 | 5023819.6 | 3.06 | 0.0014 | 1955852.6 | 2702018.1 | 1.38 | 0.0028 |
| **Protein export** | | | | | | | | | | |
| jhp1343 | YidC | putative inner membrane protein translocase component YidC; Required for the insertion of integral membrane proteins into the membrane. Probably plays an essential role in the integration of proteins of the respiratory chain complexes. Involved in integration of membrane proteins that insert dependently and independently of the Sec translocase complex (By similarity) | 258304.6 | 355192.6 | 1.38 | 0.0253 | 174144.0 | 610485.5 | 3.51 | 0.0020 |
| jhp0479 | CagV | cag island protein | 715364.7 | 1422359.2 | 1.99 | 0.0001 | 187623.4 | 1356183.4 | 7.23 | 0.0000 |
| jhp0492 | CagE | DNA transfer protein; Involved in DNA transfer. Required for induction of IL-8 in gastric epithelial cells | 199468.9 | 366421.2 | 1.84 | 0.0056 | 248512.5 | 451133.3 | 1.82 | 0.0011 |
| jhp0523 | LepB | signal peptidase I | 384774.3 | 695739.0 | 1.81 | 0.0041 | 319020.1 | 596976.9 | 1.87 | 0.0003 |
| jhp1407 | NusA | transcription elongation factor NusA; Participates in both the termination and antitermination of transcription. Interacts with RNA polymerase and binds RNA (By similarity) | 257098.2 | 396175.9 | 1.54 | 0.0000 | 506548.2 | 572235.6 | 1.13 | 0.0111 |
| jhp1126 | NusG | transcription antitermination protein NusG; Influences transcription termination and antitermination. Acts as a component of the transcription complex, and interacts with the termination factor rho and RNA polymerase (By similarity) | 1762893.2 | 3041479.9 | 1.73 | 0.0001 | 1643082.6 | 2080591.0 | 1.27 | 0.0000 |
| **Outer membrane protein** | | | | | | | | | | |
| jhp0581 | OipA | putative Outer membrane protein | 109962.5 | 165453.2 | 1.50 | 0.0057 | 69406.4 | 172225.9 | 2.48 | 0.0015 |
| jhp0238 |  | putative Outer membrane protein | 454743.8 | 604195.6 | 1.33 | 0.0027 | 385340.0 | 890038.6 | 2.31 | 0.0000 |
| jhp0073 | OMU116 | putative Outer membrane protein | 107795.9 | 200667.2 | 1.86 | 0.0021 | 99867.3 | 155761.3 | 1.56 | 0.0105 |
| jhp1394 | HopW | putative Outer membrane protein | 174537.2 | 324563.1 | 1.86 | 0.0242 | 687694.0 | 1049851.1 | 1.53 | 0.0000 |
| **Hypothetical protein** | | | | | | | | | | |
| jhp0530 |  | hypothetical protein | 2175.9 | 13313.4 | 6.12 | 0.0100 | 6327.9 | 30896.4 | 4.88 | 0.0039 |
| jhp0190 |  | hypothetical protein | 11356.1 | 69753.7 | 6.14 | 0.0093 | 17046.5 | 34075.7 | 2.00 | 0.0107 |
| jhp1095 | Csg | hypothetical protein | 6088.0 | 25216.6 | 4.14 | 0.0072 | 10403.7 | 37483.1 | 3.60 | 0.0267 |
| jhp1419 |  | hypothetical protein | 116928.7 | 243262.3 | 2.08 | 0.0289 | 89417.6 | 395414.2 | 4.42 | 0.0074 |
| jhp1457 |  | hypothetical protein | 349313.4 | 598284.6 | 1.71 | 0.0005 | 397933.6 | 1651735.4 | 4.15 | 0.0005 |
| jhp0092 |  | hypothetical protein | 59619.1 | 263822.8 | 4.43 | 0.0017 | 112415.2 | 142150.4 | 1.26 | 0.0094 |
| jhp0382 |  | hypothetical protein | 254302.2 | 610641.1 | 2.40 | 0.0020 | 98934.4 | 302773.0 | 3.06 | 0.0047 |
| jhp0932 |  | hypothetical protein | 44768.5 | 96816.8 | 2.16 | 0.0038 | 36676.9 | 86494.5 | 2.36 | 0.0001 |
| jhp0290 |  | hypothetical protein | 1072153.7 | 1603328.7 | 1.50 | 0.0004 | 1241340.3 | 3242502.4 | 2.61 | 0.0000 |
| jhp0270 |  | hypothetical protein | 120584.2 | 253308.4 | 2.10 | 0.0101 | 159717.3 | 202160.5 | 1.27 | 0.0452 |
| jhp0612 |  | hypothetical protein | 114689.0 | 280404.7 | 2.44 | 0.0441 | 188415.5 | 132382.1 | 0.70 | 0.0138 |
| jhp0261 |  | hypothetical protein | 106726.7 | 191488.1 | 1.79 | 0.0020 | 192577.0 | 223206.6 | 1.16 | 0.0209 |
